# First-line oral antiviral therapies showed similar efficacies in suppression of serum HBcrAg in chronic hepatitis B patients

**DOI:** 10.1186/s12876-021-01711-x

**Published:** 2021-03-17

**Authors:** Lung-Yi Mak, Danny Ka-Ho Wong, Ka-Shing Cheung, Wai-Kay Seto, James Fung, Man-Fung Yuen

**Affiliations:** 1 Department of Medicine, Queen Mary Hospital, The University of Hong Kong, Pokfulam Road 102,, Pok Fu Lam, Hong Kong; 2 State Key Laboratory of Liver Research, The University of Hong Kong, Pok Fu Lam, Hong Kong; 3 Department of Medicine, The University of Hong Kong-Shenzhen Hospital, Shenzhen, China

**Keywords:** Hepatitis B, Entecavir, Tenofovir, Hepatitis B core-related antigen, Nucleotide analogue

## Abstract

**Background:**

Serum hepatitis B core-related antigen (HBcrAg) is a potential surrogate marker for intra-hepatic covalently-closed circular DNA in chronic hepatitis B (CHB). We aimed to study the profiles of serum HBcrAg in CHB patients treated with first-line nucleos(t)ide analogues (NA): entecavir (ETV), tenofovir disoproxil fumarate (TDF) or tenofovir alafenamide (TAF).

**Method:**

Serum HBcrAg was measured in 120 treatment-naïve CHB patients receiving one of the 3 NAs (ETV: TDF: TAF = 60: 26: 34) using the Lumipulse G HBcrAg assay in a Lumipulse G1200 analyzer (Fujirebio Inc, Toyko, Japan). Serum HBcrAg levels were measured at week 0, week 48 and week 96 of NA therapy.

**Results:**

Among the 120 patients, 67 (55.8%) were hepatitis B e antigen (HBeAg) positive. Both tenofovir and ETV led to significantly lower serum HBcrAg at week 48 and week 96 compared to week 0. There were no significant differences for the magnitude of median HBcrAg decline at week 96 between tenofovir and ETV in HBeAg-positive (2.28 vs. 1.65 log U/mL, *p* > 0.05) and HBeAg-negative (0.83 vs. 0.54 log U/mL, *p* > 0.05) patients. TDF and TAF produced no significant differences in the magnitude of median HBcrAg decline at week 96 (HBeAg-positive: 2.63 vs. 1.83, respectively; HBeAg-negative: 1.04 vs. 0.40, respectively; both *p* > 0.05).

**Conclusion:**

Magnitude of reduction of HBcrAg levels after 2-year first-line treatment did not differ statistically among the current first-line NAs, although HBcrAg reduction was numerically greater in tenofovir-treated group. More long-term studies are essential to determine whether tenofovir exerts a more pronounced effect on HBcrAg.

**Supplementary Information:**

The online version contains supplementary material available at 10.1186/s12876-021-01711-x.

## Background

Chronic hepatitis B (CHB) infection affects 292 million worldwide and led to 0.88 million deaths as of year 2015 [1] Liver-related mortality is attributed by the development of acute on chronic liver failure, decompensated cirrhosis and hepatocellular carcinoma (HCC) [2]. In the past two decades, nucleos(t)ide analogues (NAs) have led to a major paradigm shift in the management of patients with CHB. Through suppression of viral replicative activities, with subsequent cessation of hepatic necro-inflammation and fibrosis regression [3, 4], the risk of decompensated cirrhosis, HCC and mortality can be reduced [5–7]. In the current era, 3 NAs are considered to be first-line after considering the individual efficacy, resistance profile and risk of adverse events. These include tenofovir disoproxil fumarate (TDF), tenofovir alafenamide (TAF) and entecavir (ETV), as recommended by the European Association for the Study of the Liver (EASL) and American Association for the Study of Liver Diseases (AASLD) guidelines [8, 9].

In the absence of direct head-to-head trials comparing these agents, their short-term potencies are assumed to be similar. For instance, in hepatitis B e antigen (HBeAg) positive patients, the rate of treatment induced HBeAg seroconversion at 1 year of therapy was 21%, 10% and 21% for TDF, TAF and ETV, respectively, with alanine aminotransferase (ALT) normalization rates of 68%, 72% and 68% respectively [8, 10]. However, there is emerging evidence to suggest that both short-term and long-term outcomes may differ between the various NAs. Firstly, in the phase 3 trials of TAF using TDF as a comparison, the proportion of patients with ALT normalization at week 96 was found to be higher for TAF than TDF [11]. It is postulated that the viral suppression potency is higher for TAF compared to TDF due to undefined mechanisms. Since no liver biopsies were performed in the trial, this hypothesis cannot be proven directly. Secondly, TDF has been shown to be associated with lower risk of HCC compared to ETV in a recent study and meta-analysis, while the underlying mechanisms for this phenomenon remains unknown [12, 13].

It is possible that different NAs may exert different effects on other viral replicative markers which may be important in HCC development. Higher hepatitis B core-related antigen (HBcrAg) levels, a reliable surrogate marker for intrahepatic covalently closed circular DNA (cccDNA), has been associated with higher risk of HCC in treatment-experienced CHB patients [14]. This may indicate a potential role of serum HBcrAg as a more sensitive marker in assessing the intrahepatic viral replicative activity especially in treated patients with suppressed serum HBV DNA [15]. We postulate that the 3 first-line NAs have different potency in reduction of intrahepatic cccDNA. In this study, we measured serially the serum HBcrAg as a surrogate marker of intrahepatic cccDNA, in patients treated with TDF, TAF and ETV.

## Methods

### Patients

We recruited treatment-naïve CHB patients from the Liver Clinic of Queen Mary Hospital, the University of Hong Kong, Hong Kong. All patients were seropositive for hepatitis B surface antigen for at least 6 months before treatment. Treatment-naïve patients, either HBeAg-positive or HBeAg-negative, who were newly started on NA were identified. For tenofovir, those with HBV DNA ≥ 20,000 IU/mL and serum ALT > 60 U/L (males) or > 38 U/L (females) were started on either TDF (300 mg daily) or TAF (25 mg daily). For ETV (0.5 mg daily), the indications for treatment with ETV were slightly different from tenofovir due to differences in local practice and were as follows: (1) HBeAg-positive non-cirrhotic patients with elevated ALT (upper limit of normal was defined as 50 U/L at that time) and HBV DNA > 20,000 IU/mL, or (2) HBeAg-negative non-cirrhotic patients with elevated ALT and HBV DNA > 2000 IU/mL, or (3) cirrhotic patients with detectable serum HBV DNA > 2000 IU/mL. Cirrhosis is defined by the presence of small nodular liver, splenomegaly, ascites, or varices in the porto-systemic circulation by ultrasound or computerized tomography. Patients were excluded if they were pregnant, or had concomitant hepatitis C or D virus, human immunodeficiency virus infection, Wilson’s disease, autoimmune hepatitis, primary biliary cholangitis, non-alcoholic steatohepatitis, or significant alcohol intake (20 g/day for female or 30 g/day for male). Clinical assessment and blood taking for liver biochemistry every 3–6 months were arranged for all recruited patients.

The present study was approved by the Institutional Review Board of the University of Hong Kong and the Hospital Authority Hong Kong West Cluster, Hong Kong.

### Laboratory assays

Serum HBV DNA levels were detected and quantified using Cobas Taqman assay (Roche Diagnostics, Branchburg, NJ) with a lower limit of detection (LLOD) of 10 IU/mL. For the current study, undetectable serum HBV DNA was defined as < 29 IU/mL taking reference to the same definition used in the TAF registration trial [11].

HBcrAg consists of 3 related proteins: hepatitis B core antigen, HBeAg, and a truncated 22 kDa precore protein (p22cr), which all share an identical 149 amino acid sequences. The assay has been described elsewhere [16, 17]. Briefly, particles coated with anti-HBcrAg monoclonal antibodies are added to the specimen and allowed to form antigen–antibody immune-complexes, followed by alkaline phosphatase-labelled anti-HBcrAg binding to HBcrAg of the immune-complexes on the particles which allows additional immunocomplex formation. Quantification of HBcrAg is fully automated by comparing the chemiluminescence signals generated by known concentration of recombinant ProHBeAg [15]. Serum HBcrAg were measured by the Lumipulse G HBcrAg chemiluminescence Enzyme Immunoassay (Fujirebio, Tokyo, Japan). The dynamic range of the assays ranged from 100 to 10,000,000 unit per milliliter (U/mL). Undetectable serum HBcrAg is defined as < 0.1 log U/mL. The values of HBcrAg were log transformed and were expressed in log U/mL. Serum HBcrAg levels were measured at week 0, week 48 and week 96 of NA therapy.

### Statistical analyses

Serum HBV DNA levels and HBcrAg levels were expressed in logarithm. Continuous variables were expressed in median (interquartile range). For the comparison between continuous variables, Mann–Whitney U test and Kruskal–Wallis test were used. Categorical variables were compared using Pearson’s *χ*^2^ test or Fisher’s exact test as appropriate. Spearman’s correlation coefficient was analysed to assess the relationship between serum HBcrAg and HBV DNA. Comparison between tenofovir and ETV treatment was performed. Subgroup analysis between TDF and TAF was also performed. Since HBcrAg is relatively less expressed in HBeAg-negative compared to HBeAg-positive patients, all major analyses were performed in subgroups according to the HBeAg status. All statistical analyses were performed using SPSS version 25 (SPSS, Chicago, IL). A two-sided *p* value of < 0.05 was considered statistically significant.

## Results

### Baseline

One hundred and twenty treatment-naïve CHB patients were recruited. Sixty patients (HBeAg-positive: 43 vs. HBeAg-negative: 17) were started on tenofovir and 60 patients (HBeAg-positive: 24 vs. HBeAg-negative: 36) were started on ETV. Majority of patients had elevated ALT at baseline (85.8%). The baseline characteristics were similar between tenofovir and ETV group in the HBeAg-positive patients. In the HBeAg-negative subgroup, ETV-treated patients were younger (40.9 vs. 55 years, *p* = 0.004) and had significantly higher proportion of male patients compared to tenofovir-treated patients (80.6% vs. 64.7%, *p* = 0.002). There were no significant differences of median baseline serum HBcrAg between male and female patients (3.60 vs. 3.93 log U/mL; *p* = 0.875). Due to different indications of individual antiviral as mentioned in the Methods section, none of the tenofovir-treated patients had cirrhosis compared to 11.7% of ETV-treated patients. The baseline characteristics are shown in Table 1. All 120 patients had serial measurement of serum HBcrAg at week 0, 48 and 96. None of the patients developed HBsAg seroclearance during the study period. At 96 weeks, a total of 24 patients [14/43 (32.6%) tenofovir-treated vs. 10/24 (41.7%) ETV-treated] developed HBeAg seroclearance.

**Table 1:** Baseline parameters of 120 patients

	All patients (N = 120)	HBeAg-positive (n = 67)			HBeAg-negative (n = 53)		
		TDF/TAF (n = 43)	ETV (n = 24)	*p* value	TDF/TAF (n = 17)	ETV (n = 36)	*p* value
Age (years)	42.9 (34.0–55.0)	38.0 (31.0–52.0)	43.8 (38.3–56.0)	0.136	55.0 (41.2–62.1)	40.9 (35.8–50.0)	0.004
Number of male patients	76 (63.3%)	25/43 (58.1%)	16/24 (66.7%)	0.604	11/17 (64.7%)	29/36 (80.6%)	0.002
HBcrAg (log U/mL)	3.64 (2.24–5.23)	5.23 (4.36–5.63)	4.87 (3.40–5.34)	0.066	2.03 (1.35–2.70)	2.25 (1.43–2.99)	0.517
Percentage of patients with undetectable HBcrAg*	0%	0%	0%	N/A	0%	0%	NA
ALT	73 (53–138)	79 (58–144)	89 (49–304)	0.646	57 (46–79)	72 (54–153)	0.099
Percentage of patients with elevated ALT	103/120 (85.8%)	37/43 (86.0%)	21/24 (87.5%)	1.00	16/17 (94.1%)	29/36 (80.6%)	0.412
Percentage of patients with cirrhosis	7/120 (5.8%)	0/43 (0%)	0/24 (0%)	N/A	0/17 (0%)	7/36 (19.4%)	0.082

Continuous variables were expressed as median (interquartile range)ALT: alanine aminotransferase, ETV: entecavir, HBcrAg: hepatitis B core-related antigen, HBeAg: hepatitis B e antigen, TAF: tenofovir alafenamide, TDF: tenofovir disoproxil fumarate

^*^Undetectable serum HBcrAg is defined as < 0.1 log U/mL

### Kinetics of serum HBcrAg

**Fig. 1:**
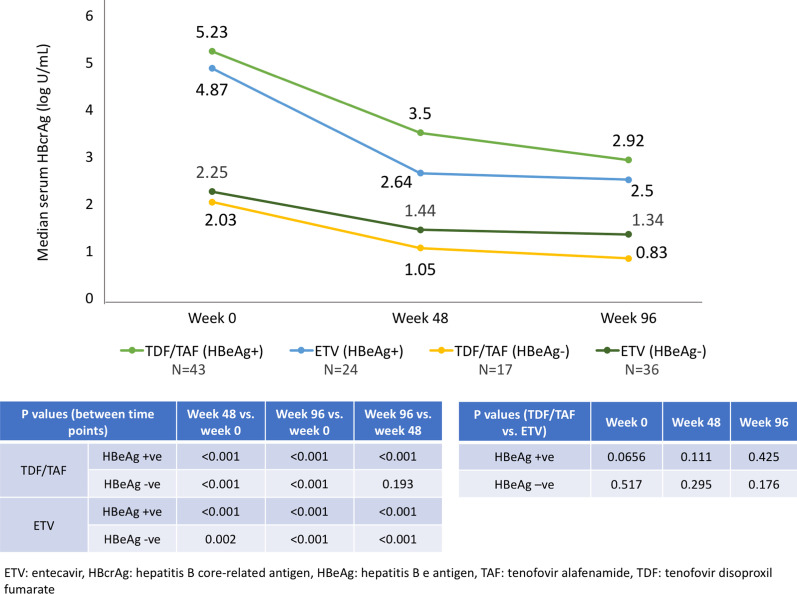
Median serum HBcrAg in different treatment groups in HBeAg-positive & HBeAg-negative patients

The median serum HBcrAg was significantly higher in HBeAg-positive patients compared to HBeAg-negative patients in both treatment groups at all 3 time points (all *p* < 0.001) (Fig. 1). In each treatment group, median serum HBcrAg was significantly lower at week 48 and week 96 compared to week 0, regardless of the HBeAg positivity (Fig. 1). There were no significant differences in the median serum HBcrAg between tenofovir-treated and ETV-treated patients at week 48 and week 96 (for HBeAg-positive patients: *p* = 0.111 and *p* = 0.425, respectively; for HBeAg-negative patients: *p* = 0.295 and *p* = 0.176, respectively).

**Fig. 2:**
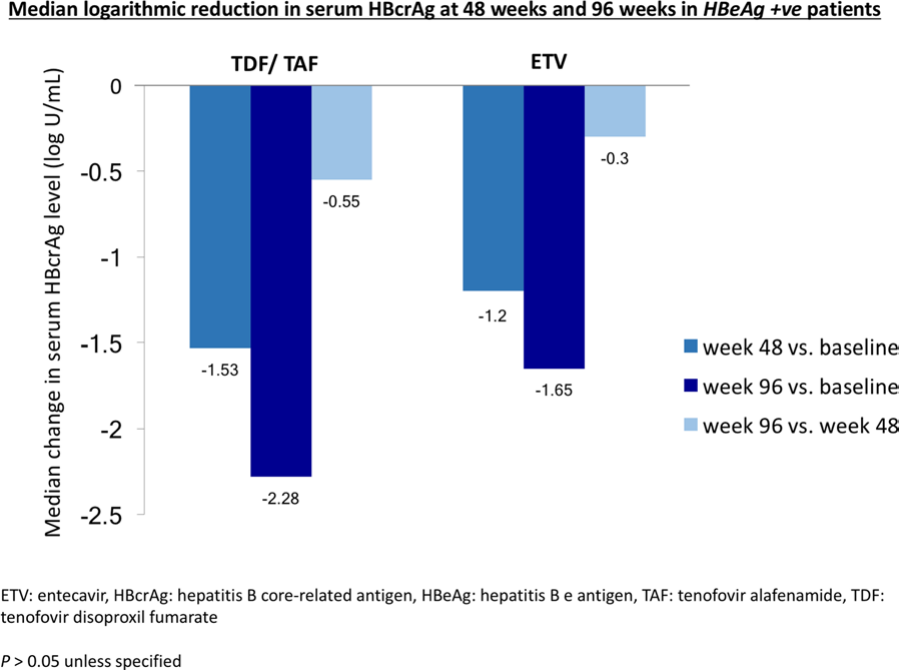
Median logarithmic reduction in serum HBcrAg at week 48 and week 96 of NA in HBeAg-positive patients

For HBeAg-positive patients, compared to week 0, tenofovir-treated patients had numerically greater decline in serum HBcrAg compared to ETV-treated patients at week 48 (1.53 vs. 1.2 log U/mL, *p* = 0.425) and week 96 (2.28 vs. 1.65 log U/mL, *p* = 0.209), although this was statistically insignificant. Compared to week 48, serum HBcrAg declined by 0.55 and 0.30 log U/mL at week 96 for tenofovir-treated and ETV-treated patients, respectively (*p* = 0.214) (Fig. 2). The degree of serum HBcrAg decline was greater in the first 48 weeks compared to the subsequent 48 weeks for both tenofovir (1.53 vs. 0.55 log U/mL, *p* > 0.05) and ETV (1.2 vs. 0.3 log U/mL, *p* > 0.05) treatment (Fig. 2).

**Fig. 3:**
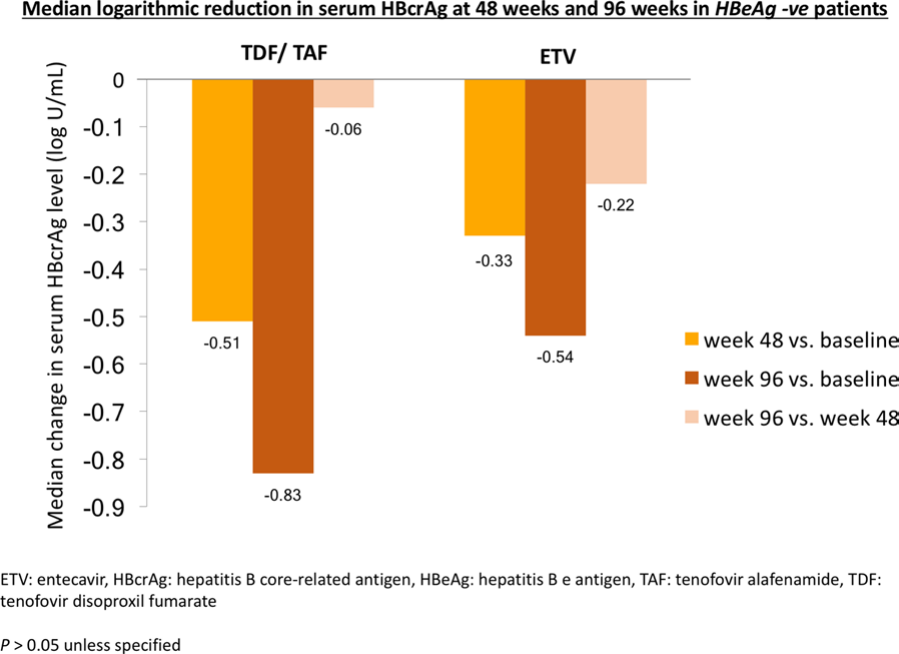
Median logarithmic reduction in serum HBcrAg at week 48 and week 96 of NA in HBeAg-negative patients

Similar to HBeAg-positive patients, for HBeAg-negative patients, compared to week 0, tenofovir-treated patients had numerically greater decline in serum HBcrAg compared to ETV-treated patients at week 48 (0.51 vs. 0.33 log U/mL, *p* = 0.391) and week 96 (0.83 vs. 0.54 log U/mL, *p* = 0.696), although again this was statistically insignificant. Compared to week 48, serum HBcrAg declined by 0.06 and 0.22 log U/mL at week 96 for tenofovir-treated and ETV-treated patients, respectively (*p* = 0.256) (Fig. 3). The degree of serum HBcrAg decline was greater in the first 48 weeks compared to the subsequent 48 weeks for both tenofovir (0.51 vs. 0.06 log U/mL, *p* > 0.05) and ETV (0.33 vs. 0.22 log U/mL, *p* > 0.05) treatment (Fig. 3). Additional analysis for the relative reduction of HBcrAg from baseline (expressed in percentage) showed similar results in term of statistically non-significant bigger magnitude of decline in tenofovir-treated patients compared to ETV-treated patients (Additional 1: Table S1).

**Fig. 4:**
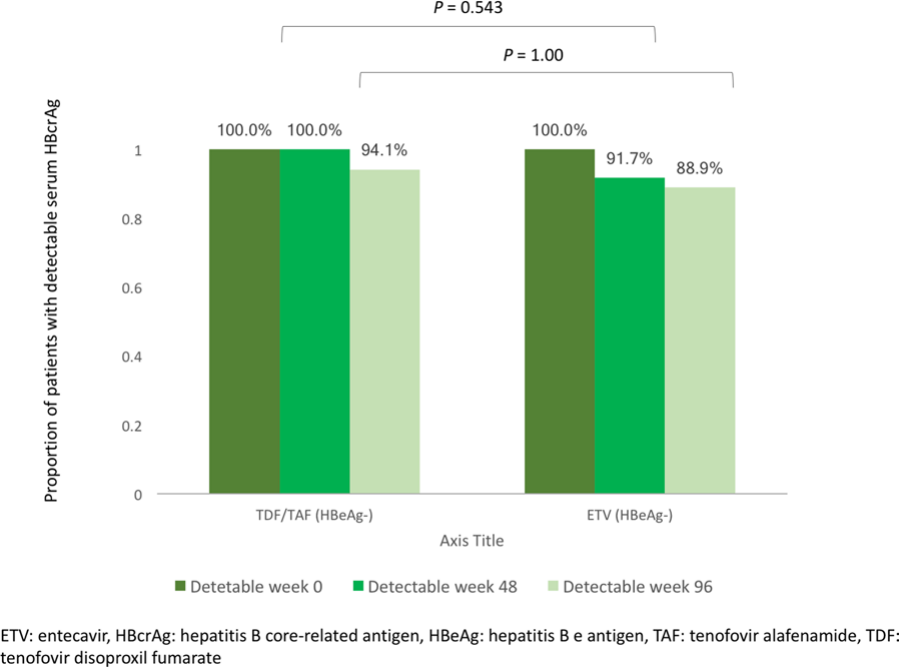
Proportion of HBeAg-negative patients with detectable serum HBcrAg

All HBeAg-positive patients had detectable serum HBcrAg at all 3 time points. In contrast, the proportion of patients with detectable serum HBcrAg declined in HBeAg-negative patients for both treatment groups (Fig. 4). At week 48, serum HBcrAg was still detectable in 100% tenofovir-treated patients and 91.7% ETV-treated patients (*p* = 0.543). At week 96, serum HBcrAg was still detectable in 94.1% tenofovir-treated patients and 88.9% ETV-treated patients (*p* = 1.00).

### Subgroup analysis of TDF vs. TAF treated patients

Twenty-six and 34 patients were treated with TDF (HBeAg-positive: negative = 18:8) and TAF (HBeAg-positive: negative = 25:9) respectively. Among tenofovir-treated HBeAg-positive patients, the median baseline serum HBcrAg were 5.37 and 5.07 log U/mL for TDF and TAF, respectively (*p* = 0.321). There were no significant differences between the median baseline serum HBV DNA between TDF and TAF (8.04 vs. 8.04 log U/mL, respectively, *p* = 0.492). Serum HBcrAg showed strong linear correlation with serum HBV DNA (r = 0.78, *p* < 0.001). Upon antiviral treatment, the decline of serum HBcrAg at week 48 and week 96 showed no statistical significance between the 2 treatment groups (Additional 1: Table S2). Serum HBcrAg still showed linear, albeit weaker, correlation with serum HBV DNA at week 48 (r = 0.425, *p* = 0.005). There was no correlation between these two viral markers at week 96 (r = 0.149, *p* = 0.366).

Among tenofovir-treated HBeAg-negative patients, the median baseline serum HBcrAg were 2.15 vs. 1.44 log U/mL for TDF and TAF, respectively (*p* = 0.301). There were no significant differences between the median baseline serum HBV DNA between TDF and TAF (5.60 vs. 5.22 log U/mL, respectively, *p* = 0.673). Serum HBcrAg showed strong linear correlation with serum HBV DNA (r = 0.80, *p* < 0.001). Upon antiviral treatment, the decline at week 48 and week 96 showed no statistical significance between the 2 treatment groups (Additional 1: Table S2). Serum HBcrAg still showed linear, albeit weaker, correlation with serum HBV DNA at week 48 (r = 0.643, *p* = 0.005). There was no correlation between these two viral markers at week 96 (r = 0.122, *p* = 0.642).

The antiviral efficacies of TDF and TAF are shown in Additional 1: Table S3. ‘ALT normalization’ was defined as patients who had ALT higher than upper limit of normal (ULN) (> 40 U/L or > 30 U/L for males and > 19 U/L for females according to the EASL and AASLD guideline respectively) [8, 9] at baseline and subsequently had normal ALT at week 48 or 96. Using the EASL definition of normal ALT, there were no significant differences in ALT normalization between TDF and TAF in both HBeAg-positive (93.8% in TDF vs. 95.2% in TAF, *p* = 0.733) and HBeAg-negative patients (85.7% in TDF vs. 87.5% in TAF, *p* = 0.833) at week 96. Similarly, using AASLD definition of normal ALT, there were no significant differences in ALT normalization between TDF and TAF in both HBeAg-positive (61.1% in TDF vs. 50.0% in TAF, *p* = 0.542) and HBeAg-negative patients (25.0% in TDF vs. 44.4% in TAF, *p* = 0.620) at week 96.

## Discussion

This study compared the antiviral efficacies in suppression of serum HBcrAg between the 3 first line NAs—TDF, TAF and ETV, in patients with HBeAg-positive chronic hepatitis or HBeAg-negative chronic hepatitis. All 3 NAs showed significant suppression of viral activity, as evidenced by serum HBcrAg reduction, which was already evident at week 48 of treatment and with continued suppression through to week 96. The rate of serum HBcrAg decline was faster during the initial 48 weeks compared to the subsequent 48 weeks, which is a consistent finding with previous report [18]. To the best of our knowledge, this is the first study in the literature that investigated the serum HBcrAg profile in TAF-treated patients, and the relative efficacy of each NA in serum HBcrAg suppression.

There was modest linear correlation between HBcrAg and HBV DNA even at 48 weeks of tenofovir treatment. No more correlation could be observed at week 96 as > 90% already had undetectable HBV DNA. Despite a high rate of achieving serum HBV DNA undetectability in both tenofovir and ETV-treated patients at 96 weeks, majority of them still had detectable serum HBcrAg (94.1% and 88.9%, respectively; *p* = 1.00). Since the rate of serum HBcrAg decline was reported to be 0.244 kU/mL/year in ETV-treated patient in a 7-year study [18], a longer treatment duration will be needed to bring down serum HBcrAg to below the lower limit of detection.

There were no significant differences in the efficacies between tenofovir and ETV, although numerically a greater decline in serum HBcrAg was observed in tenofovir-treated group. However, the current study may be under-powered to detect a significant difference due to the limited number of patients recruited in each group. In addition, it would be important to determine whether this differential reduction of HBcrAg may become significant if the comparison can be made upon more long-term treatment. The higher proportion of undetectable serum HBcrAg in ETV-treated group at week 48 and 96 (Fig. 4) may be due to the fact that there was a higher proportion of HBeAg-negative patients compared to tenofovir-treated group [36/60 (60%) vs. 17/60 (28.3%), *p* < 0.001], and is thus unlikely to reflect stronger suppression of HBcrAg by ETV. It was previously reported that serum HBcrAg is negatively correlated with age (r = −0.505, *p* < 0.001) in 139 treatment-naïve Chinese CHB patients [19], but another study reported no significant correlation (r = −0.25, *p* = 0.068) between age and serum HBcrAg in 54 similarly treatment-naïve Chinese CHB patients [16]. In the current study, the age difference between tenofovir-treated and ETV-treated groups was not statistically significant [42 (IQR 32–55) vs 43.5 (IQR 36.2–53), *p* = 0.998], and thus the effect of age on serum HBcrAg is unclear.

In a recent study of CHB patients treated with ETV and TDF for 5 years, no significant difference was observed with regards to the reduction in the risk of mortality, liver transplantation and hepatic decompensation [20]. The results of the current study somewhat provide more mechanistic insights at the fact that all 3 first-line NAs are equally effective in improving the outcomes of CHB patients. It still remains controversial whether TDF is more potent in the prevention of HCC compared to ETV. HBcrAg had been reported to be a biomarker to predict HCC development in NA-treated patients. The results from the current study did not favour tenofovir as a stronger NA compared to ETV at suppressing serum HBcrAg, which might be associated with lower risk of HCC. However, 2 years is a relatively short duration and is not able to answer this question. A longer duration of NA treatment will be necessary to observe any divergence in viral activity suppression with relevance to reduction in HCC risk.

The rate of ALT normalization at week 96 was similar between TDF and TAF in both HBeAg-positive (93.8% and 95.2%, respectively) and HBeAg-negative (85.7% and 87.5%, respectively) patients. This finding was different from a previous study which reported the 96-week outcome for the 2 phase III trials of TAF (n = 792 and n = 395 for HBeAg-positive patients and HBeAg-negative patients, respectively) [11]. This could be due to the different definition of ‘ALT normalization’ used in the current study (ALT > 40 U/L) in contrast to that used in the phase III trials (ALT > 30 U/L for male and > 19 U/L for female). Since the ULN in the current study is higher, it is relatively easier to achieve ‘normalization’ and therefore the rates were higher compared to that reported in Agarwal et al. (HBeAg-positive: 42% in TDF vs. 52% in TAF, *p* = 0.003; HBeAg-negative: 40% in TDF vs. 50% in TAF, *p* = 0.035). In the current study, if the same AASLD definition was used, the rates of ALT normalization at week 96 were similar in either HBeAg-positive patients (61.1% in TDF vs. 50.0% in TAF, *p* = 0.542) and HBeAg-negative patients (25.0% in TDF vs. 44.4% in TAF, *p* = 0.620). There were still no statistically significant differences between TDF and TAF for ALT normalization. Nevertheless, the sample size is much smaller in the current study (n = 43 and n = 17 for HBeAg-positive patients and HBeAg-negative patients, respectively). This finding is in parallel with the fact that there were no subgroup differences in terms of HBcrAg suppression between TDF and TAF.

There were several limitations in the current study. First, liver biopsies were not performed to assess the reduction in intrahepatic cccDNA and HBV DNA. However, liver biopsies may carry sampling errors and are not without risks. Nevertheless, the surrogacy of serum HBcrAg with intrahepatic cccDNA has been well described (r = 0.664–0.7) [15, 16, 21], and CHB patients may avoid this invasive procedure for the purpose of measuring cccDNA activity. Secondly, the follow-up duration was only up to 96 weeks. Long term follow-up and longitudinal measurement of serum HBcrAg would be important to ascertain the sustained virological suppression and observe any between group differences of tenofovir and ETV, in addition to the impact on improving clinical outcomes. Thirdly, the small sample size may preclude the findings of a significant difference between the treatment groups.

## Conclusion

The magnitude of reduction of HBcrAg levels after 2-year first line treatment did not differ statistically among the current first line NAs, although HBcrAg reduction was numerically greater in tenofovir treated group. More long-term treatment studies are essential to determine whether tenofovir would exert a more pronounced effect on HBcrAg.

## Supplementary Information

**Additional file 1:** Supplementary material.

## Data Availability

The dataset used and analysed during the current study are available from the corresponding author on reasonable request.

## Abbreviations

CHB: Chronic hepatitis B
HCC: Hepatocellular carcinoma
NAs: Nucleos(t)ide analogues
TDF: Tenofovir disoproxil fumarate
TAF: Tenofovir alafenamide
ETV: Entecavir
EASL: European Association for the Study of the Liver
AASLD: American Association for the Study of Liver Diseases
HBeAg: Hepatitis B e antigen
ALT: Alanine aminotransferase
HBcrAg: Hepatitis B core-related antigen
cccDNA: Covalently-closed circular DNA
