# Supplementary material for: First-line oral antiviral therapies showed similar efficacies in suppression of serum HBcrAg in chronic hepatitis B patients
Source: BMC Gastroenterol. 2021 Mar 17;21:123. doi: 10.1186/s12876-021-01711-x (PMC7968194; doi:10.1186/s12876-021-01711-x)
Supplement: Supplementary file 1 — Additional file 1: Supplementary material. [file 12876_2021_1711_MOESM1_ESM.docx]

Supplementary Table 1. Relative decline of serum HBcrAg

|  | | **Tenofovir** | **ETV** | ***P* value** |
| --- | --- | --- | --- | --- |
| HBeAg positive | Week 48 compared to week 0 | 30.9% (21.4% - 36.1%) | 28.3% (15.9% - 44.9%) | 0.734 |
|  | Week 96 compared to week 0 | 45.5% (29.6% - 55.2%) | 35.8% (21.5% - 53.4%) | 0.25 |
|  | Week 96 compared to week 48 | 10.4% (3.2% - 17.4%) | 8.2% (3.4% - 11.7%) | 0.255 |
| HBeAg negative | Week 48 compared to week 0 | 34.2% (15.7% - 66.8%) | 21.6% (-7.4% - 63.6%) | 0.322 |
|  | Week 96 compared to week 0 | 38.7% (10.5% - 74.8%) | 32.3% (5% - 66%) | 0.647 |
|  | Week 96 compared to week 48 | 3.1% (-5.6% - 18.4%) | 8.5% (1.3% - 17.9%) | 0.173 |
| ETV: entecavir, HBcrAg: hepatitis B core-related antigen, HBeAg: hepatitis B e antigen  Percentage in serum HBcrAg decline expressed as median (interquartile range) | | | | |

Supplementary Table 2. Subgroup analysis of TDF vs. TAF treatment in term of logarithmic reduction of serum HBcrAg

|  | | **TDF** | **TAF** | ***P* value** |
| --- | --- | --- | --- | --- |
| HBeAg positive | Week 48 compared to week 0 | 1.50 (0.99-2.05) | 1.57 (0.80-2.41) | 0.902 |
|  | Week 96 compared to week 0 | 2.63 (1.44-2.94) | 1.83 (1.03-2.78) | 0.228 |
|  | Week 96 compared to week 48 | 0.67 (0.26-1.19) | 0.34 (0.09-0.73) | 0.065 |
| HBeAg negative | Week 48 compared to week 0 | 1.03 (0.27-1.75) | 0.42 (0.11-1.40) | 0.321 |
|  | Week 96 compared to week 0 | 1.04 (0.22-2.14) | 0.40 (0.11-2.11) | 0.673 |
|  | Week 96 compared to week 48 | 0.05 (+0.08-0.34) | 0.06 (+0.12-0.53) | 0.963 |
| HBcrAg: hepatitis B core-related antigen, HBeAg: hepatitis B e antigen, TAF: tenofovir alafenamide, TDF: tenofovir disoproxil fumarate  Serum HBcrAg values expressed as median (interquartile range) and unit expressed as log U/mL | | | | |

Supplementary Table 3. Antiviral efficacies of TDF and TAF

|  | | **HBeAg-positive** | | | **HBeAg-negative** | | |
| --- | --- | --- | --- | --- | --- | --- | --- |
|  |  | **TDF (n=18)** | **TAF (n=25)** | ***P* value** | **TDF (n=8)** | **TAF (n=9)** | ***P* value** |
| Week 0 | HBcrAg (log U/mL) | 5.37 (4.66-5.68) | 5.07 (4.22-5.61) | 0.301 | 2.15 (1.53-2.79) | 1.44 (0.89-2.86) | 0.290 |
|  | Percentage of patients with undetectable HBcrAg* | 0% | 0% | N/A | 0% | 0% | NA |
|  | ALT (U/L) | 76 (56-147) | 81 (59-154) | 0.712 | 56 (45-103) | 57 (44-66) | 0.699 |
|  | Percentage of patients with elevated ALT by EASL criteria^ | 16/18 (88.9%) | 21/24 (87.5%) | 0.639 | 8/8 (100%) | 8/9 (88.9%) | 0.529 |
|  | Percentage of patients with elevated ALT by AASLD criteria^^ | 18/18 (100%) | 24/24 (100%) | N/A | 8/8 (100%) | 9/9 (100%) | N/A |
| Week 48 | HBcrAg (log U/mL) | 3.73 (2.80-4.08) | 3.15 (2.29-3.81) | 0.247 | 1.08 (0.81-1.71) | 0.81 (0.42-1.80) | 0.773 |
|  | Percentage of patients with undetectable HBcrAg* | 0% | 0% | N/A | 0% | 0% | N/A |
|  | ALT (U/L) | 24 (19-39) | 26 (20-45) | 0.638 | 33 (23-43) | 22 (17-38) | 0.163 |
|  | Percentage of patients with normalized ALT^ | 12/16 (75%) | 15/21 (71.4%) | 0.555 | 5/7 (71.4%) | 6/8 (75%) | 0.662 |
|  | Percentage of patients with undetectable HBV DNA** | 16/18 (88.9%) | 17/24 (70.8%) | 0.258 | 8/8 (100%) | 8/9 (88.9%) | 0.529 |
| Week 96 | HBcrAg (log U/mL) | 2.74 (2.06-3.24) | 2.92 (1.87-3.53) | 0.98 | 0.82 (0.60-1.39) | 0.83 (0.51-1.38) | 0.773 |
|  | Percentage of patients with undetectable HBcrAg* | 0% | 0% | N/A | 0% | 1/9 (11.1%) | 0.539 |
|  | ALT (U/L) | 21 (18-29) | 20 (17-28) | 0.865 | 32 (23-36) | 24 (18-35) | 0.268 |
|  | Percentage of patients with normalized ALT by EASL criteria^ | 15/16 (93.8%) | 20/21 (95.2%) | 0.722 | 6/7 (85.7%) | 7/8 (87.5%) | 0.833 |
|  | Percentage of patients with normalized ALT by 2016 AASLD criteria^^ | 11/18 (61.1%) | 12/24 (50.0%) | 0.542 | 2/8 (25.0%) | 4/9 (44.4%) | 0.620 |
|  | Percentage of patients with undetectable HBV DNA** | 18/18 (100%) | 19/22 (86.4%) | 0.243 | 8/8 (100%) | 8/9 (88.9%) | 0.529 |
| ALT: alanine aminotransferase, HBcrAg: hepatitis B core-related antigen, HBeAg: hepatitis B e antigen, TAF: tenofovir alafenamide, TDF: tenofovir disoproxil fumarate  *Undetectable serum HBcrAg is defined as <0.1 log U/mL  **Undetectable serum HBV DNA is defined as <29 IU/mL  ^ The upper limit of normal is 40 U/L; elevated ALT is defined as >40 U/L ^8^  ^^ The upper limit of normal is 30 U/L for male and 19 U/L for female; elevated ALT is defined as >30 U/L for male and >19 U/L for female ^9^  Continuous variables are expressed as median (interquartile range) | | | | | | | |
